# Identification of Cyclopropaneoctanoic Acid 2-Hexyl in Human Adipose Tissue and Serum

**DOI:** 10.1007/s11745-013-3806-2

**Published:** 2013-06-11

**Authors:** Tomasz Sledzinski, Adriana Mika, Piotr Stepnowski, Monika Proczko-Markuszewska, Lukasz Kaska, Tomasz Stefaniak, Julian Swierczynski

**Affiliations:** 1 Department of Pharmaceutical Biochemistry, Medical University of Gdansk, ul. Debinki 1, 80-211 Gdansk, Poland; 2 Department of Molecular Evolution, Faculty of Biology, University of Gdansk, ul. Wita Stwosza 59, 80-308 Gdansk, Poland; 3 Department of Environmental Analysis, Faculty of Chemistry, University of Gdansk, ul. Sobieskiego 18/19, 80-952 Gdansk, Poland; 4 Department of General, Endocrine and Transplant Surgery, Medical University of Gdansk, ul. Smoluchowskiego 17, 80-214 Gdansk, Poland; 5 Department of Biochemistry, Medical University of Gdansk, ul. Debinki 1, 80-211 Gdansk, Poland

**Keywords:** Cyclopropane fatty acids, Cyclopropaneoctanoic acid 2-hexyl, Cyclopropaneoctanoic acid 2-octyl, Cyclopropanenonanoic acid, 2-[[2-[(2-ethylcyclopropyl)methyl]cyclopropyl]methyl] acid, Fatty acids content, Human adipose tissue

## Abstract

Fatty acids containing a cyclopropane ring in their structure (cyclopropane FA) have been found in a wide variety of bacteria, a number of protozoa, and Myriapoda. Little is known about cyclopropane FA in mammal, especially in human tissues. The present study deals with the identification of cyclopropane FA in adipose tissue and serum of humans and rats. Fatty acids extracted from the adipose tissue and serum obtained from obese women during bariatric surgery were methylated and analyzed on GC–MS. We have identified: cyclopropaneoctanoic acid 2-hexyl, cyclopropaneoctanoic acid 2-octyl, cyclopropanenonanoic acid, and 2-[[2-[(2-ethylcyclopropyl)methyl]cyclopropyl]methyl] acid in human adipose tissue. We confirmed the presence of cyclopropaneoctanoic acid 2-hexyl by derivatization of FA extracted from human adipose tissue to picolinyl esters. Cyclopropaneoctanoic acid 2-hexyl was the main cyclopropane FA (approximately 0.4 % of total fatty acids in human adipose tissue, and about 0.2 % of total fatty acids in the serum). In adipose tissue cyclopropaneoctanoic acid 2-hexyl was found mainly in triacylglycerols, whereas in serum in phospholipids and triacylglycerols. The cyclopropaneoctanoic acid 2-hexyl has also been found in serum, and adipose tissue of rats in amounts comparable to humans. The content of cyclopropaneoctanoic acid 2-hexyl decreased in adipose tissue of rats maintained on a restricted diet for 1 month. In conclusion, we demonstrated that cyclopropaneoctanoic acid 2-hexyl is present in human adipose tissue and serum. Adipose tissue cyclopropaneoctanoic acid 2-hexyl is stored mainly in triacylglycerols and the storage of this cyclopropane FA is affected by food restriction.

## Introduction

Adipose tissue (AT) constitutes the main depot of energy stored as triacylglycerols (TAG) in the human body. In the last two decades, AT has been studied extensively as an endocrine organ, producing and releasing many biologically active proteins and peptides, called adipokines [1]. However, free fatty acids (FFA) originating from hydrolysis of TAG are released by AT in the greatest amount [1]. FFA released from AT circulate in the blood complexed with albumin and are collected by organs (mainly skeletal and heart muscle, kidney cortex) as a substrate for energy production. Elevated serum FFA concentrations (a state, which is often present in obese subjects) lead to several abnormalities including insulin resistance, inflammatory responses, and a decrease in NO production [1]. It is believed that the quality of dietary fat, rather than its quantity, have an impact on these abnormalities, especially with regards to insulin sensitivity [2]. A number of fatty acids (FA), which could be stored and released by AT, have beneficial effect on health [3, 4], whereas others have been associated with detrimental consequences [5, 6]. Recently, 17:1n-7 palmitoleic acid was identified as an AT-derived lipid hormone (a lipokine) that stimulates insulin action in mice muscle and suppresses hepatosteatosis in mice [7, 8]. Collectively, the above presented studies show increased interest in the regulatory function of FA released by AT and inspire further search for potentially biologically active FA stored and released by AT.

Cyclopropane FA contain three-carbon carbocyclic rings located at different sites of FA chain. They have been found in plants, bacteria, parasites, sponges and *Ascidia* [9–11]. Bao et al. [12] identified a gene encoding cyclopropane synthase catalyzing the addition of methylene group from *S*-adenosylmethionine to the double bond of oleic acid in phospholipids of *Sterculia foetida*. Wood and Reiser [13], after feeding rats with diet containing cyclopropane FA (food containing 0.54 % of methyl *cis*-9,10-methylene octanodecanoate and methyl trans-9,10-methylene octanodecanoate), have found these cyclopropane FA in AT of rats. In a more recent paper Sakurada et al. [14] identified *cis*-9,10-methylenehexadecanoic acid (also called cyclopropaneoctanoic acid 2-hexyl) in phospholipids of human, rat, and bovine heart, as well as in human and rat liver. FA containing cyclopropane rings could display biological activity. For instance, 2-hexyl-cyclopropanedecanoic acid increased human cyclooxygenase activity [15]. In guinea pig myocardium, *cis*-9,10-methylenehexadecanoic acid inhibited the activity of actomyosin ATPase [16]. Kanno et al. [17] showed that a synthetic derivative of linoleic FA containing two cyclopropane rings, 8-[2-(2-pentylcyclopropylmethyl)-cyclopropyl]-octanoic acid (DCP-LA) selectively activated PKC-ɛ. Recently, this group reported the positive effect of DCP-LA on the age related learning and memory deterioration in mice [18].

The purpose of this study was to use readily available AT, obtained from obese women during bariatric surgery, to test hypothesis whether human AT stores cyclopropane FA. Moreover, we also examined whether cyclopropane FA are present in adipose tissue and blood of rats fed a standard laboratory diet and rats maintained on a restricted diet for 1 month. To the best of our knowledge, this is the first time that data presented here indicate that: (a) cyclopropaneoctanoic acid 2-hexyl is present in human and rat AT and serum, (b) cyclopropaneoctanoic acid 2-hexyl is mainly stored as a component of TAG in human AT, (c) cyclopropaneoctanoic acid 2-hexyl content in rat AT is affected by food restriction, (d) other cyclopropane FA (cyclopropaneoctanoic acid 2-octyl, cyclopropanenonanoic acid, and 2-[[2-[(2-ethylcyclopropyl)methyl]cyclopropyl]methyl]) were detected in small amounts (up to 0.05 % of total FA) in AT of some patients, and were undetectable in human serum.

## Materials and Methods

### Adipose Tissue and Blood of Obese Patients

Sixteen non-diabetic, obese women (mean age 43 ± 11 years) underwent a Roux-en-Y gastric bypass (RYGB) at the Department of General, Endocrine, and Transplant Surgery (Medical University of Gdansk, Poland). The inclusion criteria consisted of the absence of clinical evidence of endocrine, cardiac, hepatic, or renal diseases. Smokers were excluded from the study. After an overnight fast, blood specimens were collected and centrifuged to obtain serum samples.

The patients’ fat mass was measured with the Tanita SC 330S Body composition Analyzer. Standard laboratory parameters were assayed by the Central Clinical Laboratory of the Medical University of Gdansk. During the surgery, pieces of visceral and subcutaneous AT, weighing approximately 1 g each, were removed and immediately frozen in liquid nitrogen. The tissues and serum were stored at −80 °C until further analysis was performed. The investigations were approved by the Medical University of Gdansk Ethics Committee (protocol number NKEBN/208/2010) and were conducted within the framework of world medical association declaration of Helsinki. All patients participating in the study signed an informed, written consent form. The selected anthropometric and laboratory parameters of 16 studied women are presented in Table 1.

**Table 1:** Characteristics of patients included in the study

Parameter	Mean ± SD
BMI (kg/m^2^)	43 ± 8.8
Body weight (kg)	117 ± 31
Adipose tissue mass (kg)	48 ± 21
Total serum cholesterol (mg/dL)	153 ± 32
Serum triacylglycerols (mg/dL)	113 ± 48
Serum glucose (mg/dL)	95 ± 12
Serum insulin (μU/ml)	7.8 ± 5.4
HOMA	1.9 ± 1.5
Total serum protein (g/L)	68 ± 6.7
Serum bilirubin (mg/dL)	0.36 ± 0.20
Serum creatinine (mg/dL)	0.77 ± 0.21

*BMI* body mass index, *HOMA* homeostasis model assessment score

### Adipose Tissue and Blood of Rats

Ten-week old male Wistar rats, weighing approximately 240 g at the onset of the experiment, housed in individual wire-mesh cages, were maintained at 22 ^o^C under a light to dark (12/12 h) cycle with lights on at 7:00 a.m. The rats were divided randomly into 2 groups. Control animals (*n* = 10) were allowed free access to food and tap water. The remaining group (*n* = 10) were allowed free access to tap water and obtained 50 % of the total amount of food consumed by the control group for 1 month. Food was replenished every day, 2 h before the lights off period. The average daily food intake of the control rats (rats fed ad libitum) was approximately 26 g throughout the period of the experiment. The commercial diet used in all groups was the same as described previously [19]. After the treatment, the rats were anaesthetized with ketamine (60 mg/kg of body mass) and xylazine (6 mg/kg of body mass) administration and killed. Blood specimens from rats were collected and centrifuged to obtain serum samples. The pieces of AT were removed and immediately frozen in liquid nitrogen. The tissue and serum were stored at −80 °C until further analysis was performed. The rat intestinal content was obtained from colons of control rats. The study was consistent with the EU Directive 2010/63/EU for animal experiments and was approved by the Local Ethics Committee for Experimental Animals in Gdansk, Poland (protocol number 14/2012).

### GC–MS Analysis of Fatty Acids

The total lipids were extracted according to Folch et al. [20]. The lipid samples (obtained from 0.2 g of AT or 0.5 mL of serum) were hydrolyzed with 1 mL of 0.5 M KOH in methanol at 90 °C for 3 h. The mixture was acidified with 0.2 mL of 6 M HCl and then 1 mL of water was added. FFA were extracted three times with 1 mL of *n*-hexane, and evaporated to dryness in a stream of nitrogen. FA methyl esters (FAME) were prepared using 1 mL of 10 % boron trifluoride reagent (BF_3_/methanol) at 55 °C for 90 min. One milliliter of water was added to the reaction mixture and FAME were extracted three times with 1 mL of *n*-hexane and the solvent was evaporated. To prepare the picolinyl esters of FA, unesterified FA were dissolved in 0.5 mL of trifluoroacetic anhydride and left for 30 min at 50 °C. The excess of reagent was blown off in a stream of nitrogen. Next, 0.2 mL of dichloromethane containing 18 μg of 3-hydroxymethylpyridine and 4 mg of 4-dimethylaminopyridine were added. The mixture was left for 3 h at room temperature; afterwards, the solvent was removed in a stream of nitrogen and washed with 8 mL of *n*-hexane and 4 mL of water. Subsequently, the mixture was mixed. The picolinyl esters of FA were extracted three times with 8 mL of *n*-hexane and solvent was evaporated.

Both FAME and picolinyl FA esters were analyzed with GC-EI-MS QP-2010 SE (Shimadzu) similarly as described previously [21–24]. FA esters were separated on a 30 m × 0.25 mm i.d., HP-5 capillary column (film thickness 0.25 μm). The column temperature was programmed from 60 to 300 °C for FAME separation, and 200 to 300 °C for picolinyl ester separation at a rate of 4 °C/min with helium as the carrier gas at a column head pressure of 60 kPa. For ionization of both FAME and picolinyl FA esters, the electron energy was 70 eV. The internal standard was 19-methyl-eicosanoate. Chemicals and reagents were obtained from Sigma-Aldrich. Based on the: (a) standards, (b) mass spectra of FA and (c) data published, the individual FA were identified. Additionally, library NIST 2010 (National Institute of Standards and Technology) and GC–MS Solution Version 2.70 (Shimadzu Corporation) were used to confirm the results of the identification.

### Separation of FFA, TAG, and Phospholipids

The lipids extracts prepared from human AT and serum according to Folch et al. [20] were separated into FFA, TAG, and phospholipids using high performance liquid chromatography with a laser light scattering detector (HPLC-LLSD) and a normal-phase 250 × 4.6 mm analytical column packed with Econosil Silica (Alltech, particle size 5 μm). The mobile phase consisted of *n*-hexane (Solvent A) and dichloromethane containing 15 % acetone (Solvent B). The gradient was programmed linearly from A to B within 35 min. After the separation, TAG and phospholipids (PL) were subjected to hydrolysis by the method described above. Next, FAME were prepared from FFA and FA originating from separated TAG and PLs, and analyzed by GC–MS as described above.

### Statistical Analysis

The statistical significance of differences between the groups was assessed by a one-way analysis of variance (ANOVA) and Tukey’s post hoc test used for further determination of significance of differences. Differences between the groups were considered significant when *p* < 0.05. All data are presented as means ± SD. Sigma Stat software was used for all statistical analyses.

## Results

**Fig. 1:**
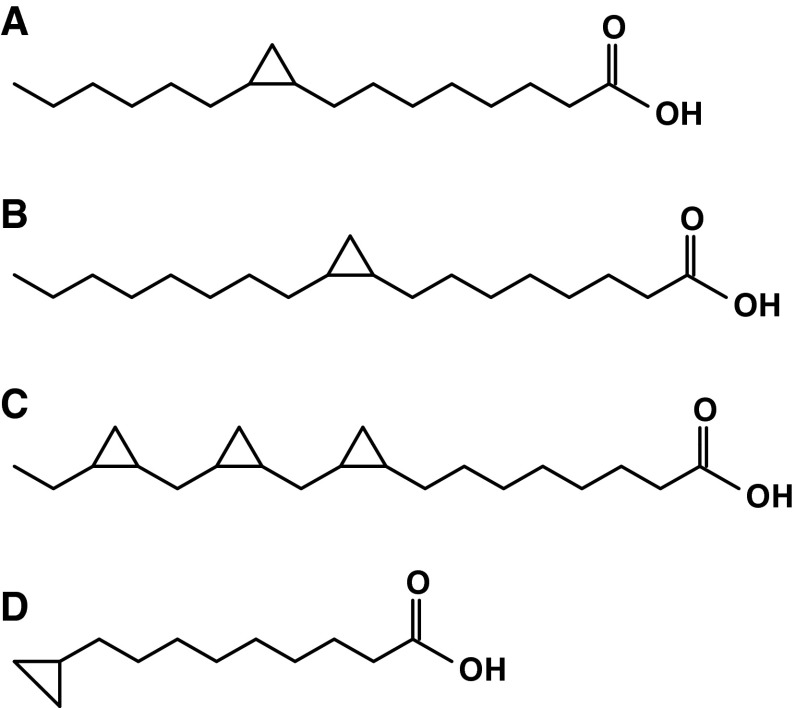
Chemical structure of cyclopropane fatty acids identified in human adipose tissue: cyclopropaneoctanoic acid 2-hexyl (**a**), cyclopropaneoctanoic acid 2-octyl (**b**), 2-[[2-[(2-ethylcyclopropyl)methyl]cyclopropyl]methyl] acid (**c**), and cyclopropanenonanoic acid (**d**)

**Fig. 2:**
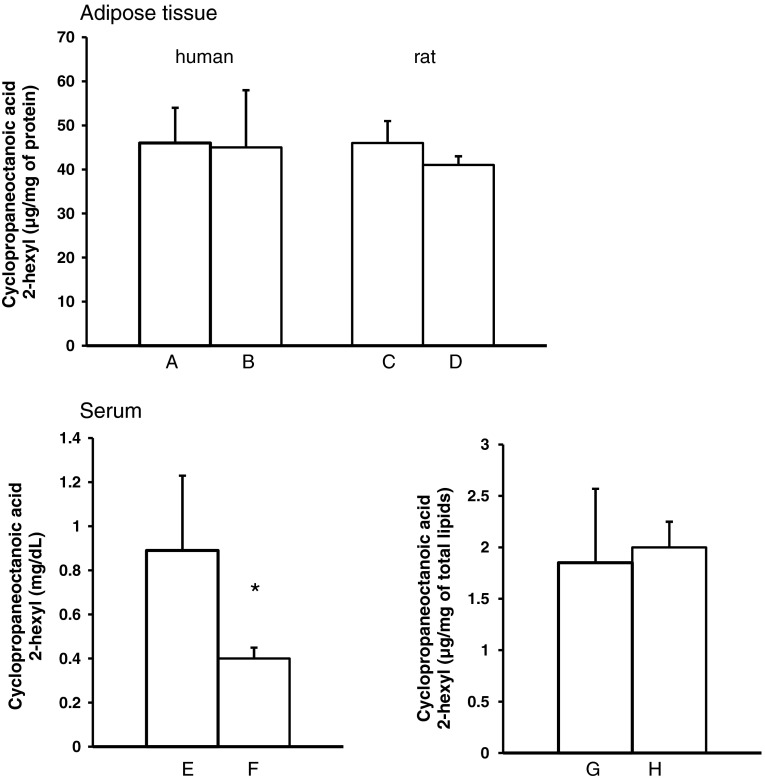
Cyclopropaneoctanoic acid 2-hexyl content of visceral (**a**) and subcutaneous (**b**) adipose tissue of human, visceral (**c**) and subcutaneous (**d**) adipose tissue of rat and serum of human (**e**, **g**) and rat (**f**, **h**). Data are presented as means ± SD. * *p* < 0.05

**Fig. 3:**
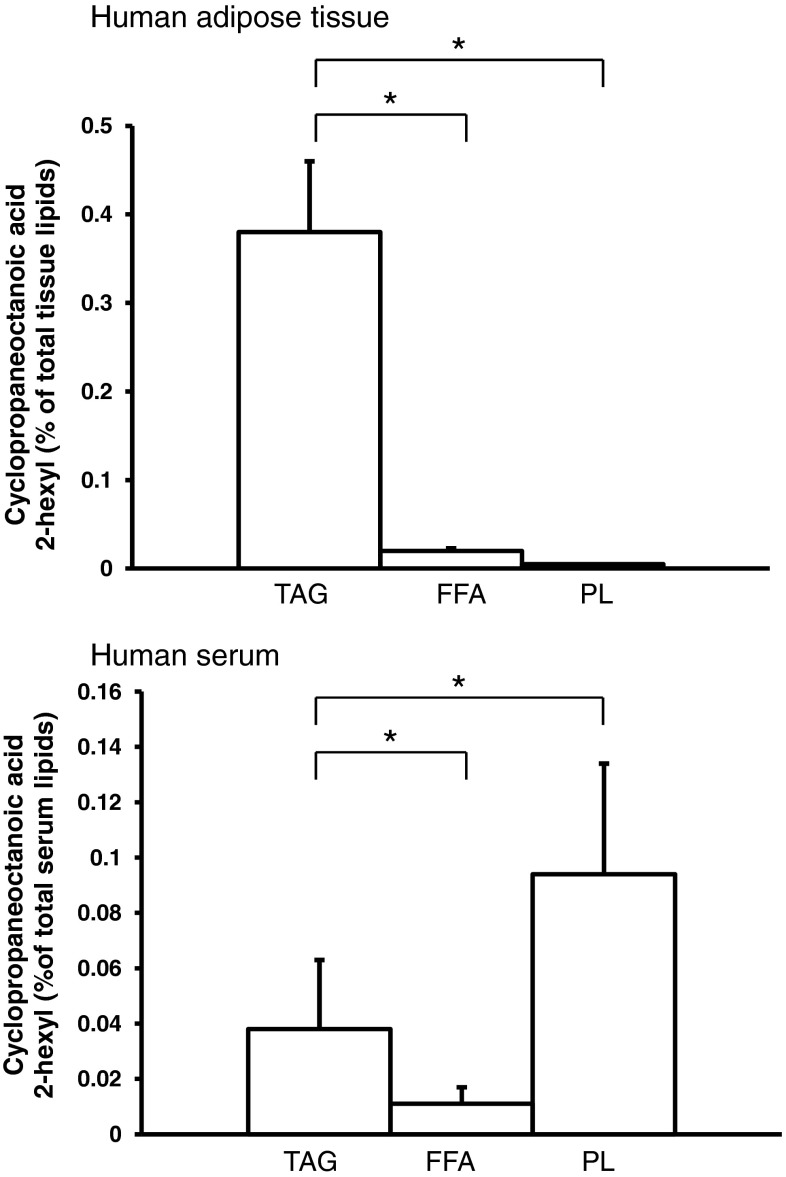
Cyclopropaneoctanoic acid 2-hexyl amounts in triacylglycerols (TAG), free fatty acids (FFA), and phospholipids (PL) of human visceral adipose tissue and serum. Data are presented as means ± SD. *p < 0.05

The amounts of FA expressed as proportions of the total FA of human serum, visceral, and subcutaneous AT are presented in Table 2. The major FA in visceral and subcutaneous AT were MUFA, followed by SFA and PUFA. Palmitic acid was the major SFA, followed by the stearic acid. Oleic acid was the major MUFA and linoleic acid was the major PUFA. Overall, the most abundant FA in human AT was oleic acid, followed by palmitic acid and linoleic acid. Proportions of FA were essentially similar in visceral and subcutaneous AT as well as in the serum (Table 2). Moreover, in human AT, we have identified cyclopropaneoctanoic acid 2-hexyl (Fig. 1a), cyclopropaneoctanoic acid 2-octyl (Fig. 1b), 2-[[2-[(2-ethylcyclopropyl)methyl]cyclopropyl]methyl] acid (Fig. 1c), and cyclopropanenonanoic acid, (Fig. 1d). Cyclopropaneoctanoic acid 2-hexyl (Fig. 1a) was the main FA containing a cyclopropane ring. It accounts for approximately 0.4 % of the total FA in human AT, both from the visceral and subcutaneous depots, and about 0.2 % of the total FA in the serum (Table 2). The other cyclopropane FA were detected in small amounts (up to 0.05 % of total FA) in AT of some patients (8 of 16 examined), and were undetectable in human serum (Table 2). Cyclopropaneoctanoic acid 2-hexyl has also been found in visceral (Fig. 2c) and subcutaneous (Fig. 2d) AT as well as in rat serum (Fig. 2f, h). The amounts of cyclopropaneoctanoic acid 2-hexyl in rat visceral (Fig. 2c) and subcutaneous (Fig. 2d) AT were comparable with human visceral (Fig. 2a) and subcutaneous AT (Fig. 2b) when expressed in μg per mg of AT protein. The concentrations of cyclopropaneoctanoic acid 2-hexyl (expressed in mg per dL) in human serum (Fig. 2e) were approximately twofold greater compared to rat serum (Fig. 2f). This was due to an approximately twofold higher concentration of lipids in human serum compared to rat serum [25]. When cyclopropaneoctanoic acid 2-hexyl was expressed as μg per mg of lipids, the amount of this cyclopropane FA was essentially similar in humans and rats (Fig. 2g, h). The data presented in Fig. 3 indicate that cyclopropaneoctanoic acid 2-hexyl extracted from human visceral adipose tissue is a component of TAG. Human serum cyclopropaneoctanoic acid 2-hexyl is present mainly in phospholipids and TAG (Fig. 3).

**Table 2:** Fatty acids composition of visceral and subcutaneous adipose tissue and serum of obese patients

Fatty acid	Visceral adipose tissue fatty acids (% ± SD)	Subcutaneous adipose tissue fatty acids (% ± SD)	Serum fatty acids (% ± SD)
14:0 Myristic	2.8 ± 0.76	2.8 ± 0.59	1.2 ± 0.5
16:0 Palmitic	21.2 ± 1.7	22.7 ± 0.94	24.5 ± 2.5
18:0 Stearic	5.2 ± 0.94	5.1 ± 1.2	6.2 ± 0.7
Other SFA	1.9 ± 0.53	1.7 ± 0.48	1.6 ± 0.32
Total SFA	31.1 ± 2.8	32.3 ± 2.0	33.6 ± 2.2
16:1 Palmitoleic	7.0 ± 1.3	6.0 ± 1.7	3.8 ± 1.0
18:1 Oleic	37.1 ± 4.0	39.1 ± 4.3	34.5 ± 4.0
20:1 Eicosenoic	2.1 ± 0.37	1.8 ± 0.43	0.43 ± 0.30
Other MUFA	0.84 ± 0.21	0.60 ± 0.21	0.23 ± 0.13
Total MUFA	47.1 ± 3.2	47.7 ± 3.8	38.9 ± 4.9
18:2 Linoleic	18.3 ± 3.4	16 ± 2.7	18.6 ± 3.1
20:4 Arachidonic	0.41 ± 0.12	0.59 ± 0.22	4.6 ± 1.9
Total n-6 PUFA	20.6 ± 3.6	18.7 ± 3.1	25.1 ± 4.7
Total n-3 PUFA	0.68 ± 0.24	0.85 ± 0.32	2.0 ± 0.93
Total PUFA	21.3 ± 3.6	19.5 ± 3.2	27 ± 5.5
**Cyclopropaneoctanoic 2-hexyl**	**0.40** **±** **0.072**	**0.39** **±** **0.11**	**0.19** **±** **0.071**
Cyclopropaneoctanoic 2-octyl	0.032 ± 0.007	0.038 ± 0.012	ND
Cyclopropanenonanoic	0.014 ± 0.005	0.017 ± 0.006	ND
2-[[2-[(2-Ethylcyclopropyl)methyl] cyclopropyl]methyl]	TR	TR	ND

*ND* not detected, *TR* trace amounts (<0.01 %)

Cyclopropaneoctanoic acid 2-hexyl is the main cyclopropane FA in human adipose tissue and serum (which is indicated in bold)

Considering that cyclopropane FA are produced by intestinal bacteria [26], theoretically they could be absorbed from the digestive tract into the circulation, transported to AT and, ultimately stored in human AT. Moreover, some authors suggest that intestinal flora are different in lean and obese subjects [27–29]. Thus, one would expect an association between the BMI of patients and the cyclopropane FA content in AT or serum. We have found no such association (*r* = 0.06, NS in visceral AT; *r* = −0.05, NS in subcutaneous AT). However, further studies with greater numbers of lean and obese subjects are needed to confirm these findings.

**Fig. 4:**
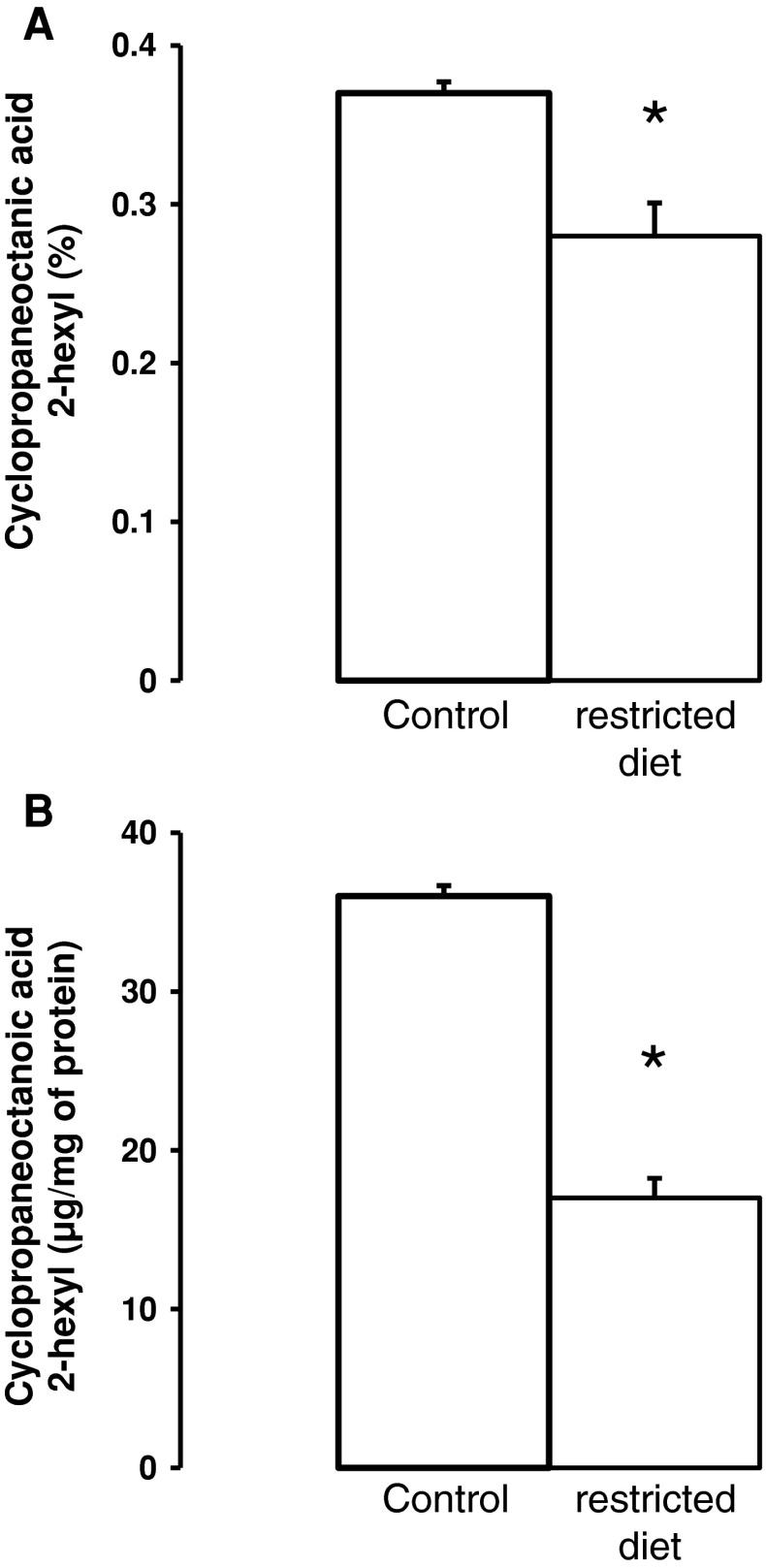
Cyclopropaneoctanoic acid 2-hexyl content of adipose tissue of control rats and rats maintained on a restricted diet for 1 month. Data are expressed as percentages of cyclopropaneoctanoic acid 2-hexyl composition of adipose tissue (**a**) or as μg of cyclopropaneoctanoic acid 2-hexyl per mg of adipose tissue protein **b**. Data are presented as means ± SD. *p < 0.05

Data presented in Fig. 4a indicate that, in visceral AT of rats maintained on a restricted diet for 1 month, the level of cyclopropaneoctanoic acid 2-hexyl decreased by about 20 %. When the data are expressed as μg of cyclopropaneoctanoic acid 2-hexyl per mg of AT protein, the decrease in rats maintained on restricted diet reached approximately 50 % of the control value.

**Fig. 5:**
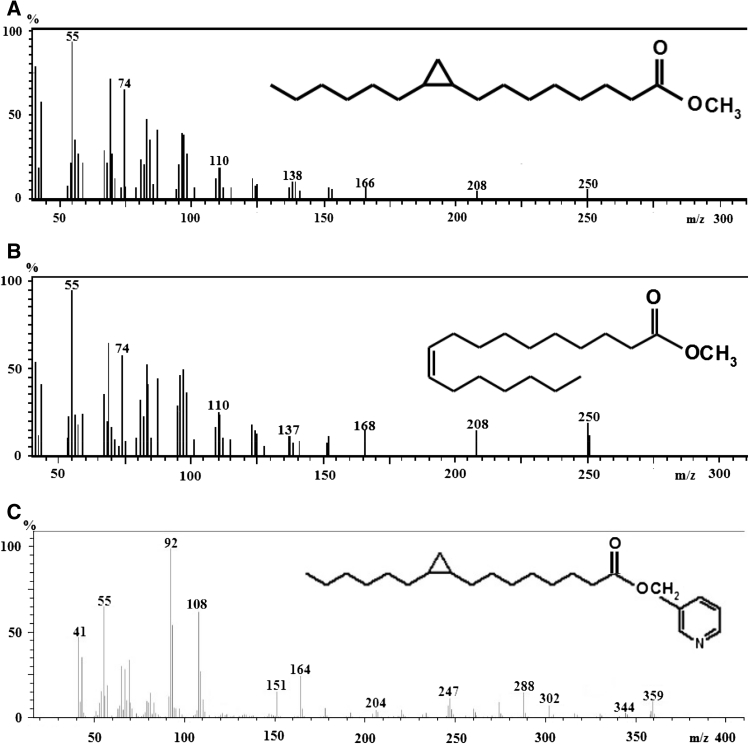
The structure and electron ionization mass spectra of cyclopropaneoctanoic acid 2-hexyl methyl ester (**a**), *cis*-10-heptadecenoic acid methyl ester (**b**) and cyclopropaneoctanoic acid 2-hexyl picolinyl ester (**c**)

Unfortunately, cyclopropaneoctanoic acid 2-hexyl, the major FA containing cyclopropane ring found in human and rat AT and serum (Fig. 5a) and *cis*-10-heptadecenoic acid (Fig. 5b) have the same retention time (RT), and very similar electron ionization MS. To rule out the possibility that *cis*-10-heptadecenoic acid was erroneously identified as cyclopropaneoctanoic acid 2-hexyl, commercially available *cis*-10-heptadecenoic acid methyl ester was analyzed under the same conditions as FAME from AT or serum. The *cis*-10-heptadecenoic acid standard was correctly identified by library NIST 2010. The main FA containing cyclopropane ring present in AT or serum was identified as cyclopropaneoctanoic acid 2-hexyl. Therefore, these experiments excluded the possibility that *cis*-10-heptadecenoic acid was mistakenly identified as cyclopropaneoctanoic acid 2-hexyl. Moreover to confirm the presence of the cyclopropaneoctanoic acid 2-hexyl in human adipose tissue we prepared cyclopropaneoctanoic acid 2-hexyl picolinyl esters. Figure 5c shows the mass spectra of the cyclopropaneoctanoic acid 2-hexyl picolinyl ester. The typical ions of a picolinyl esters are in the lower molecular weight region at *m*/*z* 92, 108, 151 (McLafferty rearrangement) and 164 (Fig. 5c, [30]). The ion at *m*/*z* 247 is the characteristic fragment in the spectrum of the cyclopropane picolinyl ester, which allows to identify the presence of the cyclopropane FA and its position in the acyl chain [21, 30]. The ion is produced by cleavage through the cyclopropane ring and represents a fragment containing carbon 9 in the cyclopropane ring, together with the remainder of the molecule on the same side as the picolinyl ester group. Ions at *m*/*z*344, 330, 316, 302, 288 and 274 originate from the cleavage between successive methylene groups and ions at *m*/*z* 220, 206, 192, 178 and 164 are from the cleavage between methylene group on the carboxyl group side. Moreover, mass spectrum presented in Fig. 5c shows the same fragment ions as a mass spectrum for cyclopropaneoctanoic acid 2-hexyl picolinyl ester (3-pyridylcarbinyl 9,10-methylene-hexadecanoate) published in [31]. These results seem to be conclusive proof of the presence of cyclopropaneoctanoic acid 2-hexyl in human AT.

In theory, cyclopropaneoctanoic acid 2-hexyl could be formed (non-enzymatically) by the methylation of double bond of palmitoleic acid, which is present in human AT (Table 2) during the experimental procedure according to reaction: palmitoleic acid + CH_3_OH → methyl-hexadecanoic acid → cyclopropaneoctanoic acid 2-hexyl (by analogy to enzymatic methylation catalyzed by cyclopropane synthase [11]). Thus, we performed an additional experiment to exclude such a possibility. Commercially available palmitoleic acid was methylated at the identical conditions as FA extracted from AT or serum and analyzed. The GC–MS analysis showed only palmitoleic acid in this sample. Cyclopropaneoctanoic acid 2-hexyl was not detected under these conditions. The same procedure was performed with oleic acid (which is also present in human AT, Table 2) to exclude the formation of cyclopropaneoctanoic acid 2-octyl. Again, no cyclopropane FA was detected in this sample. Therefore, this experiment excluded the possibility of the formation of cyclopropane FA during preparation of FAME. Collectively, the results presented above support the conclusion that human AT stores cyclopropane FA and cyclopropaneoctanoic acid 2-hexyl is the main cyclopropane FA stored as a component of TAG.

To answer the question about the origin of cyclopropaneoctanoic acid 2-hexyl in AT and serum, we analyzed the FA composition of rat laboratory food (looking for cyclopropane FA in food) and rat intestinal content (looking for cyclopropane FA produced by intestinal bacteria). Unfortunately using the same procedure for cyclopropane FA extraction and detection as for AT and serum, we were unable to detect any cyclopropane FA in rat laboratory food or in rat intestinal content (not shown). Moreover, cyclopropaneoctanoic acid 2-hexyl content in blood from the portal vein was slightly lower than in blood from the inferior vena cava (not shown). The opposite results (i.e. higher content of cyclopropaneoctanoic acid 2-hexyl in the portal vein than in the vena cava) could be expected assuming that cyclopropane FA originate from food and/or are produced by intestinal bacteria.

## Discussion

In this paper, we have shown for the first time that cycloproane FA (Fig. 1) are present in human AT and serum (Table 2). In human visceral and subcutaneous AT the level of cyclopropaneoctanoic acid 2-hexyl, the main FA containing cyclopropane ring, was essentially similar to the level of arachidonic acid (Table 2), which plays an essential role in many physiological processes, being a precursor of prostaglandins, thromboxanes and leukotrienes [32]. The AT cyclopropaneoctanoic acid 2-hexyl was found mainly in TAG (Fig. 3) of all patients examined in this study. Since TAG are the major lipids in AT, the presence of cyclopropaneoctanoic acid 2-hexyl mainly in TAG in AT was expected. The presence of cyclopropane FA in serum is not surprising as it is generally believed that the plasma FFA pool is determined by FA release from AT [33]. In a number of subjects three other cyclopropane FA in AT were identified (Fig. 1), however, in at least 10-times lower amounts (Table 2). This low amount of cyclopropane FA in human AT is probably the reason why we were unable to detect these cyclopropane FA in serum of all patients included in the study. The presence of cyclopropaneoctanoic acid 2-hexyl in AT and serum suggest that AT can take up and release cyclopropane FA into the circulation. The storage of cyclopropane FA in AT may protect other organs from exposure to excessive cyclopropane FA. However, cyclopropane FA derived from AT (during active lipolysis) could influence the function of other organs.

It is generally believed that human visceral and subcutaneous AT have a different metabolism and turnover [34]. Thus, one would expect different amounts of cyclopropaneoctanoic acid 2-hexyl in these AT depots. The data presented here indicate that this is not the case (Fig. 2a, b).

Until now, only Sakurada et al. [14] has reported the presence of cyclopropane FA, namely cyclopropaneoctanoic acid 2-hexyl (also called *cis*-9,10-methylenehexadecanoic acid), in phospholipids from human tissues—heart and liver, as well as in heart and liver of some mammals. Wood and Reiser [13] have found cyclopropane FA in rat AT, after feeding animals for 5 weeks with food containing cyclopropane FA. To the best of our knowledge, no data on the presence of cyclopropane FA in human AT and serum have been reported so far. Moreover, no data on the presence of cyclopropane FA in the AT of rats fed a standard laboratory diet or rats maintained on a restricted diet have been reported. Thus, it was of the highest importance to exclude the possibility that our results could constitute an artifact, resulting from: (a) incorrect identification of other FA as a cyclopropane FA, and (b) formation of cyclopropane FA during the preparation of the sample for analysis. To test the first possibility (incorrect identification of some FA), we searched for FA, whose methyl ester has a similar (or even identical) RT and electron ionization MS to methylated cyclopropaneoctanoic acid 2-hexyl. We found that *cis*-10-heptadecenoic acid methyl ester has the same RT and very similar MS (Fig. 5a, b). The experiments performed here excluded the possibility of erroneous identification of *cis*-10-heptadecenoic acid as cyclopropaneoctanoic acid 2-hexyl. Unequivocal proof of the presence of cyclopropaneoctanoic acid 2-hexyl in human AT was established through synthesis of cyclopropaneoctanoic acid 2-hexyl picolinyl ester (Fig. 5c).

To test the possibility of the formation of cyclopropaneoctanoic acid 2-hexyl during the experimental procedures, FA that could, theoretically, be transformed to this cyclopropane FA was sought. In plants and bacteria, cyclopropane FA could be formed by the methylation of double bond in unsaturated FA followed by cyclization of methyl group by cyclopropane synthase [11]. Thus, one can suppose that palmitoleic acid present in AT or serum can be non-enzymatically (chemically) methylated during FAME formation (before GC–MS analysis), and further cyclized to cyclopropaneoctanoic acid 2-hexyl. Methylation of palmitoleic acid under the same conditions as FA from AT or serum, followed by GC–MS analysis showed no cyclopropaneoctanoic acid 2-hexyl in methylated sample of palmitoleic acid. Thus, we believe that these results exclude the possibility of the formation of cyclopropane FA during our experimental procedures.

Finally, the question arises about the source of cyclopropaneoctanoic acid 2-hexyl (and other cyclopropane FA) in human AT and serum. In theory, three possibilities have to be taken into consideration. First, cyclopropane FA may originate from food. However, we have not detected cyclopropaneoctanoic acid 2-hexyl in lipids extracted from rat laboratory food. Second, cyclopropaneoctanoic acid 2-hexyl could originate from intestinal bacteria [13]. To check this hypothesis we analyzed FA composition (using the same procedure for cyclopropane FA extraction and detection as for tissue and serum) of the rat intestinal content. Again, we were unable to detect cyclopropaneoctanoic acid 2-hexyl in the rat intestinal content. However, it cannot be excluded that cyclopropane FA are present in food and are produced by intestinal bacteria at a low level, below the limit of detection. Thus, it is likely that cyclopropane FA, even if consumed and/or produced by intestinal bacteria at a very low level (below the limit of detection by the procedure used), can accumulate in AT. Finally, cyclopropaneoctanoic acid 2-hexyl acid could be synthesized by some organ/tissue in human and rat body. As already mentioned, until now the activity of cyclopropane synthase has been identified only in plants, bacteria, and parasites [11]. However, it cannot be excluded that such reactions could occur in mammal tissues. At present, it is difficult to establish the source of cyclopropane FA in human AT. Considering that the FA composition of AT is a reliable biomarker for long-term dietary intake of FA [33], one can suppose that the main source of cyclopropane FA in AT is the consumed food. Even if ingested at low levels, mainly in the form of plants, dairy products, and ruminant (beef) meat, cyclopropane FA could accumulate in AT and be released into circulation. However, further research is needed to establish unequivocally the main source of cyclopropane FA in human AT. The presence of cyclopropaneoctanoic acid 2-hexyl acid both in the blood and AT suggests that this cyclopropane FA can be taken up and released by AT (adipocytes).

Data presented here indicate also that in AT of rats maintained on a restricted diet for 1 month, the level of cyclopropaneoctanoic acid 2-hexyl significantly decreased. These results suggest that physiological changes in rats can significantly influence the level of cyclopropane FA in rat AT. It is likely that similar changes are taking place in human AT. One can suppose that the decrease in the cyclopropaneoctanoic acid 2-hexyl level in adipose tissue of rats maintained on a restricted diet, supports the hypothesis that cyclopropane FA originate from food.

The previously described examples of biological activity of cyclopropane FA [15–18] together with the results presented here suggest that cyclopropaneoctanoic acid 2-hexyl could have some regulatory function in human AT. Moreover, the presence of this FA in subcutaneous and visceral AT suggests that cyclopropaneoctanoic acid 2-hexyl could be released by human AT, and if some biological activity of this FA would be confirmed it could be defined as a lipokine. Further research is needed to verify these hypotheses.

At first, during the process of searching for cyclopropane FA in human AT, it was necessary to study the FA composition in AT and serum of subjects included in the study. The most abundant FA in human AT was oleic acid, followed by palmitic acid, and linoleic acid (Table 2). The data presented here indicate that the human FA composition of AT is essentially similar to those reported by Hernandes-Morante et al. [35] in obese patients, and Iggman et al. [36].

In conclusion, the main finding reported in this paper is the identification of cyclopropaneoctanoic acid 2-hexyl in human AT and serum. The presence of cyclopropaneoctanoic acid 2-hexyl as a component of TAG in AT and serum (as a FFA and a component of TAG and phospholipids) suggests that AT is able to take up and release cyclopropaneoctanoic acid 2-hexyl into the circulation. The storage of cyclopropane FA in AT may protect other organs from exposure to excessive cyclopropane FA. However, cyclopropane FA derived from AT (during active lipolysis) could influence the function of other organs. We found for the first time the presence of cyclopropane FA in AT and serum of rats fed a standard laboratory diet. Moreover, we found a decrease in cyclopropaneoctanoic acid 2-hexyl in AT of rats maintained on restricted diet. Taken together, the results presented here and published previously indicate that cyclopropane FA are present not only in bacteria, plants, protozoa, and Myriapoda but also in mammalian tissues including humans. At present, we can anticipate that the results presented here will be the starting point for more complex studies aimed at gaining more information about the source and a possible pathophysiological significance of cyclopropane FA accumulation in human AT.

## Abbreviations

CLA: Conjugated linoleic fatty acids
DCP-LA: 8-[2-(2-pentylcyclopropylmethyl)-cyclopropyl]-octanoic acid
FAME: Fatty acids methyl esters
MS: Mass spectra
PKC-ɛ: Protein kinase C-ɛ
RT: Retention time
RYGB: Roux-en-Y gastric bypass
